# Metastatic Brain Tumors Disrupt the Blood-Brain Barrier and Alter Lipid Metabolism by Inhibiting Expression of the Endothelial Cell Fatty Acid Transporter Mfsd2a

**DOI:** 10.1038/s41598-018-26636-6

**Published:** 2018-05-29

**Authors:** Shweta Tiwary, John E. Morales, Sam C. Kwiatkowski, Frederick F. Lang, Ganesh Rao, Joseph H. McCarty

**Affiliations:** Department of Neurosurgery, The University of Texas M. D. Anderson Cancer Center, Houston, TX 77030 USA

## Abstract

Disruption of the blood-brain barrier (BBB) by cancer cells is linked to metastatic tumor initiation and progression; however, the pathways that drive these events remain poorly understood. Here, we have developed novel patient-derived xenograft (PDX) models of brain metastases that recapitulate pathological growth features found in original patient samples, thus allowing for analysis of BBB disruption by tumor cells. We report that the BBB is selectively disrupted in brain metastases, in part, via inhibition of the endothelial cell-expressed docosahexaenoic acid (DHA) transporter, major facilitator superfamily domain 2a (Mfsd2a). Loss of Mfsd2a expression in the tumor endothelium results in enhanced BBB leakage, but reduced DHA transport and altered lipid metabolism within metastases. Mfsd2a expression in normal cerebral endothelial cells is cooperatively regulated by TGFβ and bFGF signaling pathways, and these pathways are pathologically diminished in the brain metastasis endothelium. These results not only reveal a fundamental pathway underlying BBB disruption by metastatic cancer cells, but also suggest that restoring DHA metabolism in the brain tumor microenvironment may be a novel therapeutic strategy to block metastatic cell growth and survival.

## Introduction

Each year in the USA more than 200,000 people are diagnosed with metastatic brain cancer^1^. Brain metastasis is a common complication in patients with advanced primary lung cancer, breast cancer, and melanoma, with 50% of lung and melanoma patients and approximately 20% of breast cancer patients developing secondary lesions in the brain^2^. Studies in each of these cancers reveal common cell-intrinsic pathways as critical drivers of metastatic potential to the brain. For example, loss of PTEN, which activates the PI3K-AKT pathway, correlates with significantly increased risk of brain metastasis in melanoma^3^. Similarly, the PTEN pathway is suppressed in metastatic cells by astrocytes in the brain microenvironment through exosomal-delivered miRNAs that inhibit PTEN expression, thus promoting tumor growth and survival^4^. Alternations in stromal components of the brain microenvironment are also essential for continued tumor growth and progression. Metastatic tumor cells upregulate various extracellular proteases such as cathepsins that promote extravasation from blood vessels and enable early stages of perivascular growth^5^. In addition, tumor cell-induced alterations in the cerebral vasculature via suppression of the plasmin pathway drive metastatic seeding and growth^6^. The exchange of factors via gap junctions between brain cancer cells and resident astrocytes protect tumors from chemotherapy^7^. Hence, it is necessary to understand how metastatic cells co-opt stromal components in the brain microenvironment for selective growth and survival.

A common feature in most brain metastases is resistance to therapy, which is attributed to the poor penetration of therapeutics across the BBB. Very little is understood about pathways that control BBB permeability in the normal brain or in brain tumors, and these gaps in knowledge impede the potential to exploit the BBB for drug delivery^8^. This lack of knowledge is due, in part, to a dearth of animal models that accurately recapitulate tumor pathophysiology. Many animal models of brain metastases rely heavily on mouse and human cell line variants that have been grown in culture for decades^9^. These metastatic models, although useful for studying tumor cell homing to the brain, do not fully mimic many of the microenvironmental pathologies observed in patients with brain metastases. For example, commonly used models of melanoma metastasis give rise to encapsulated, perivascular lesions in the mouse brain. Leptomeningeal dissemination occurs in many patients with brain metastases from breast cancer, although these growth patterns are uncommon in many mouse models. Hence, there is a clear need for pre-clinical models that reproduce pathophysiological growth features, including critical alterations to the brain microenvironment, observed in patients.

MFSD2a is a nutritionally regulated gene with important roles in mammalian tissue and organ growth, lipid metabolism and cognitive and motor functions^10^. In the brain and retina Mfsd2a selectively transports the omega-3 fatty acid DHA across the BBB, with genetic deletion of Mfsd2a protein in mice leading to impaired DHA transport and reduced levels of vital lipid metabolites^11,12^. Loss-of-function familial mutations in human MFSD2A are linked to cognitive deficits and ataxia due to deficiencies in DHA transport and metabolism^13,14^. In addition to mediating transport of DHA, Mfsd2a suppresses caveolin-dependent transcytosis, with genetic deletion of murine Mfsd2a leading to enhanced transcellular transport and breakdown of the vascular endothelial barrier in the brain^15^ and retina^16^. Here, we have generated a panel of novel patient-derived xenograft (PDX) mouse models of brain metastases to study signaling pathways involved in disruption of the intratumoral BBB. We show that Mfsd2a expression as well as its transport functions are selectively down regulated in the metastatic brain tumor vascular endothelium. This down-regulation is due to the absence of astrocytes that normally maintain expression of Mfsd2a in cerebral endothelial cells through TGFβ1 and bFGF signaling. Loss of MFSD2A promotes metastatic tumor growth and survival in the brain microenvironment by altering DHA transport and metabolism, revealing that restoring DHA and/or its metabolites to the tumor microenvironment may be an effective treatment strategy for patients with metastatic brain cancer.

## Results

**Figure 1:**
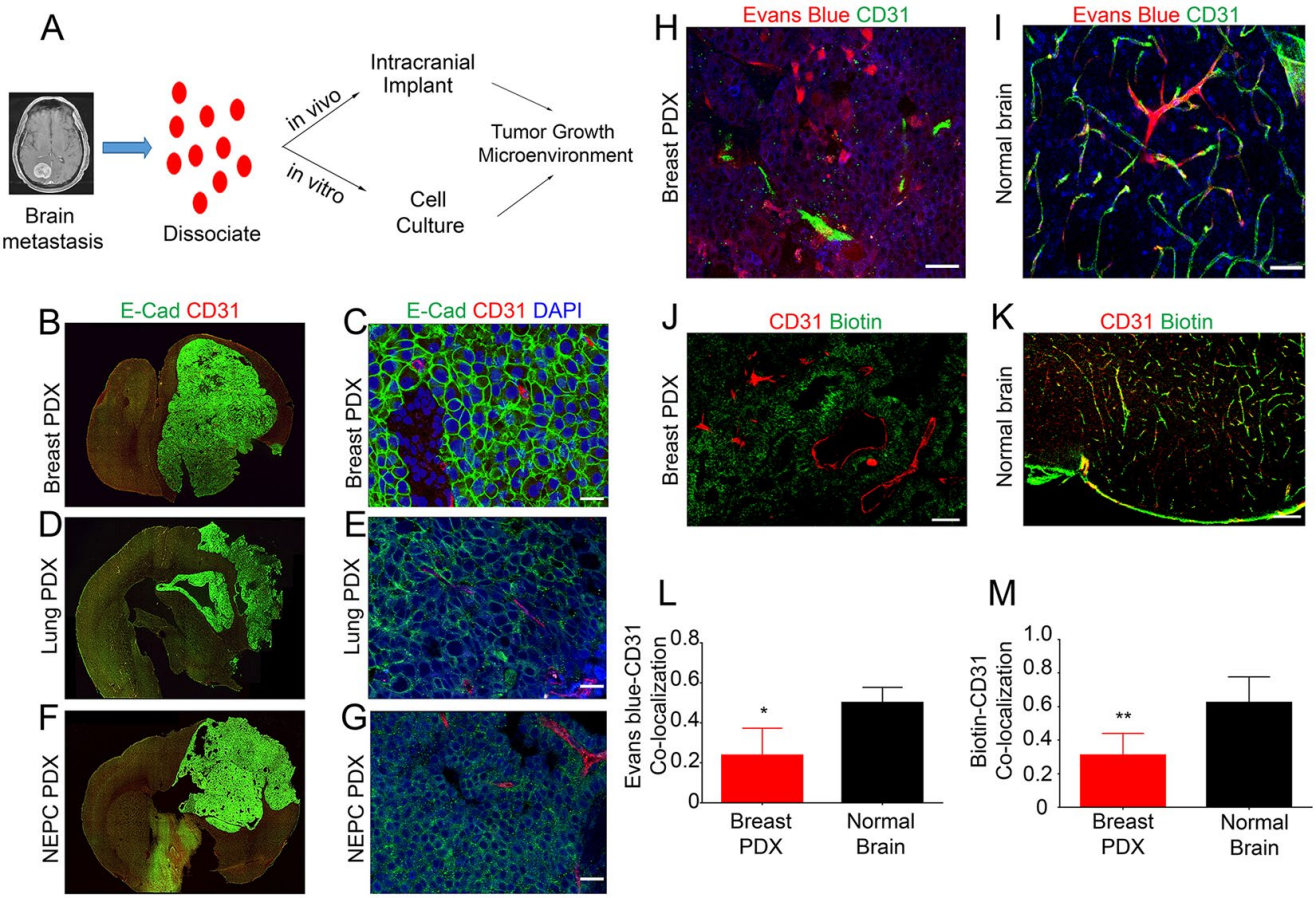
Development of PDX models of brain metastasis and analysis of BBB permeability defects *in vivo*. (**A**) Summary of strategies for culturing primary metastatic cells *in vitro* and establishing PDX models of brain metastases using freshly resected human tumor specimens. (**B**–**G**) Representative immunofluorescence images showing E-cadherin expression in PDX metastatic brain tumors derived from human breast cancer (**B**,**C**) lung cancer (**D**,**E**), and NEPC (**F**–**I**) Representative fluorescent images of coronal brain slices after injection of Evans blue (EB) tracer showing extravasation from CD31-expressing blood vessels and accumulation in the microenvironment of a breast cancer brain metastasis (**H**), in comparison to the non-cancerous mouse brain hemisphere showing retention of EB in the cerebral circulation (**I**) Representative images of mouse brains showing extravasation of amine-reactive biotin after cardiac perfusion. Note the pathological leakage across the BBB in PDX models of brain metastases derived from breast cancer (**J**) versus the non-cancerous brain hemisphere (**K**,**L**) Quantitation of EB extravasation in breast cancer brain metastasis tissue versus normal brain parenchyma, *p < 0.05. (**M**) Quantitation of amine-reactive biotin extravasation in PDX models of breast cancer brain metastasis as compared to non-cancerous mouse brain tissue, **p < 0.005. All scale bars are 100 μm.

To analyze how metastatic brain tumor cells interact with stromal components in the neural microenvironment, we set out to establish a panel of primary cultured cells and PDX mouse models using freshly resected metastatic brain tumor tissues derived from primary tumors that frequently home to brain, including breast (n = 3), lung (n = 4), and neuroendocrine prostate cancer (NEPC, n = 1). Cultured metastatic tumor cells grew as neurosphere-like spheroids in serum-free media and expressed epithelial markers including E-Cadherin and β-catenin (Supplemental Figure S1). To generate PDX tumor models, freshly prepared cell suspensions from metastatic tissues or low-passage cultured cells were injected into the striata of NOD-SCID mice (Fig. 1A). Patient-derived metastatic cells generated large, non-invasive intracranial tumors that showed epithelial-like features including expression of E-cadherin (Fig. 1B–G and Supplemental Figures S2 and S3). Similar membranous E-cadherin expression was observed in sections of human breast cancer brain metastasis, as determined by immunofluorescence staining (Supplemental Figure S4).

To analyze the functional integrity of the BBB in brain metastases *in vivo*, we monitored vascular permeability after injecting tumor-bearing mice with Evan’s Blue (EB) or amine-reactive biotin (sulfo-NHS-biotin), which do not normally cross the intact BBB^17,18^. EB is normally retained in the circulation in a complex with serum albumin, a 65 kDa protein. Sulfo-NHS-biotin is approximately 500 Daltons and normally conjugates to free amines of proteins found on the luminal side of blood vessels. Analysis of fixed brain sections revealed a disrupted BBB, as indicated by EB (Fig. 1H) and sulfo-NHS-biotin (Fig. 1J) extravasation from blood vessels into the tumor parenchyma. In contrast, analysis of the opposite non-injected brain hemisphere, which did not contain metastatic tumor, revealed EB (Fig. 1I) and sulfo-NHS-biotin (Fig. 1K) retention within the lumens of blood vessels. Interestingly, analysis of the fluorescent tracer extravasation patterns in breast cancer brain metastasis models revealed blood vessel-like structures that lacked a CD31-expressing lining endothelium (Fig. 1H,J). Hence, Breast cancer brain metastasis cells appear to form CD31-negative channels that may connect to the functional intratumoral vasculature. Quantitative analysis of tracer leakage by measuring fluorescent signal in the parenchyma surrounding CD31-expressing blood vessels revealed a significant increase in permeability of blood vessels within metastatic tissue compared to the non-injected hemisphere (Fig. 1M). Analysis of fixed human sections from breast cancer brain metastases also revealed a heterogeneously disrupted BBB, based on extravasation of circulating human IgG protein (Supplemental Figure S5). We did not detect obvious differences in the expression of tight junction proteins in intratumoral endothelial cells (Supplemental Figure S6). However, tumor cells expressed tight junction protein components normally present in the endothelial BBB. For example, VE-Cadherin and Claudin3 proteins were detected in metastatic tumor cells (Supplemental Figure S6). In addition, we detected expression of the glucose transporter Glut1 in metastatic tumor cells. Expression of these junction proteins demonstrates that tumor cells may induce the formation of a “brain-tumor barrier” that could account for the refractory nature of brain metastases to therapies.

**Figure 2:**
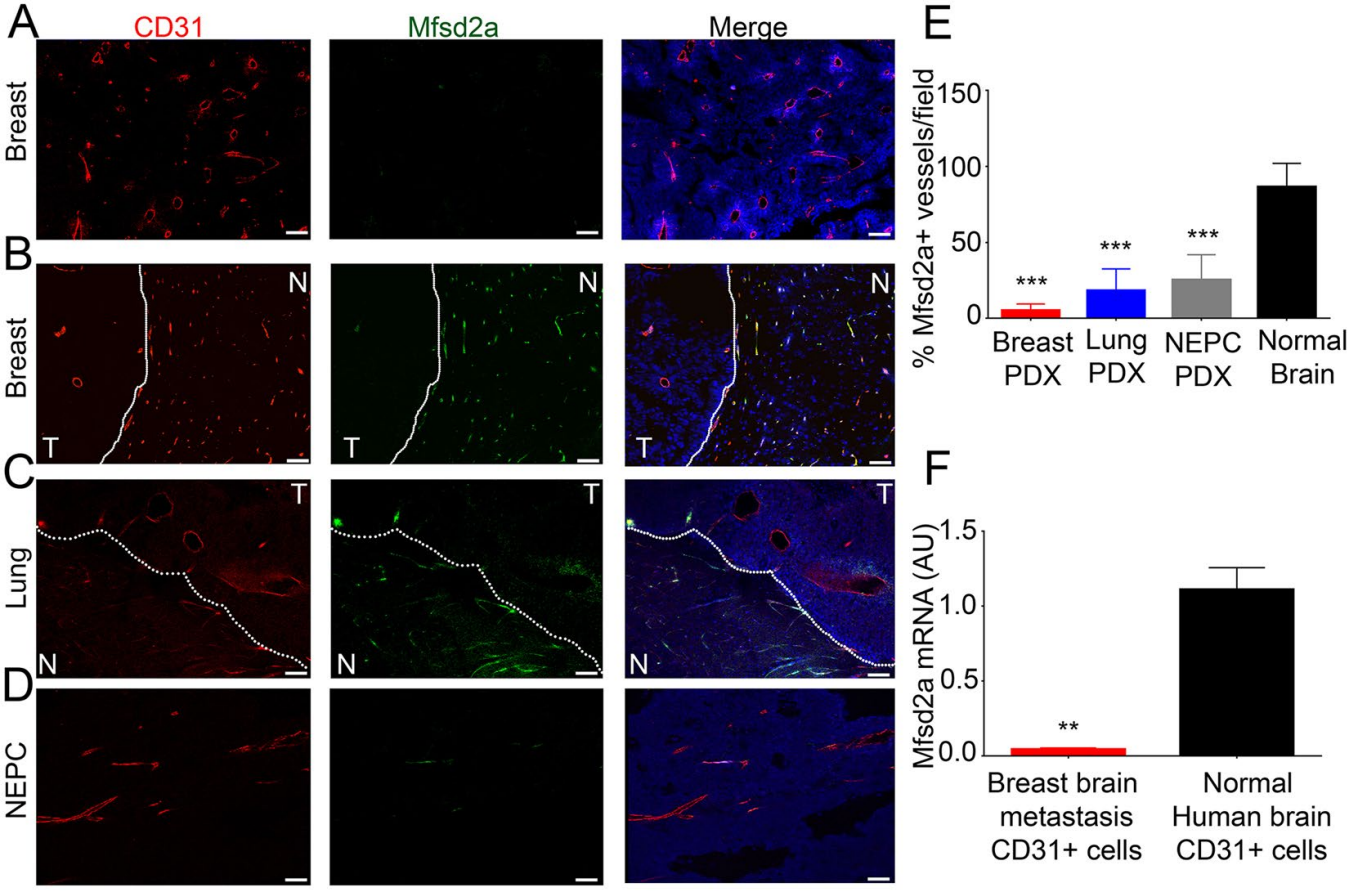
*In vivo* analysis of Mfsd2a protein expression in brain metastasis endothelial cells. (**A**,**B**) Anti-Mfsd2a and anti-CD31 double immunofluorescence labeling of fixed sections from PDX mouse models of breast cancer brain metastasis (T, tumor) showing adjacent non-cancerous mouse brain tissue (N, normal). Note the Mfsd2a protein expression in the normal brain vasculature, with marked reduction of Mfsd2a expression in the metastatic brain tumor vasculature (**A**,**B**). The dashed line indicates the tumor margin. (**C**,**D**) Double immunofluorescence staining of PDX models of brain metastases derived from lung cancer (**C**) and NEPC (**D**) reveal decreased Mfsd2a protein levels in the CD31-expressing tumor endothelium. The dashed line in (**C**) marks tumor margins. (**E**) Quantitation of Mfsd2a expression in the CD31^+^ vascular endothelium of PDX models of brain metastasis, as compared to non-cancerous mouse brain. Note the marked reduction in Mfsd2a protein levels in tumor endothelial cells, ****p < 0.001. (**F**) Quantitation of human MFSD2A mRNA levels in CD31^+^ vascular endothelial cells fractionated from a breast brain metastasis in comparison to endothelial cells fractionated from non-cancerous human brain tissue. Note the significant reduction in MFSD2A mRNA levels in the metastatic tumor endothelial cell fractions, **p < 0.01. All scale bars are 100 μm.

Mfsd2a was recently reported to regulate BBB properties in cerebral endothelial cells^11^. Since intratumoral blood vessels showed abnormal BBB properties, we analyzed the expression of Mfsd2a in metastatic tumors as well as in the contralateral (opposite) non-injected hemisphere. Our data showed a significant decrease of Mfsd2a protein expression in vascular endothelial cells (Fig. 2E) within brain metastases originating from primary breast cancer (Fig. 2A,B), lung cancer (Fig. 2C), and NEPC (Fig. 2D) patient samples, as revealed by double immunofluorescence labeling with anti-Mfsd2a and anti-CD34 antibodies. Consistent with the immunohistochemical data from the PDX models of metastases, we also detected significant down-regulation of MFSD2A mRNA in CD31^+^ vascular endothelial cells fractionated from freshly resected human breast cancer brain metastases, as compared to CD31^+^ cells fractionated from adjacent non-cancerous brain tissue (Fig. 2F).

**Figure 3:**
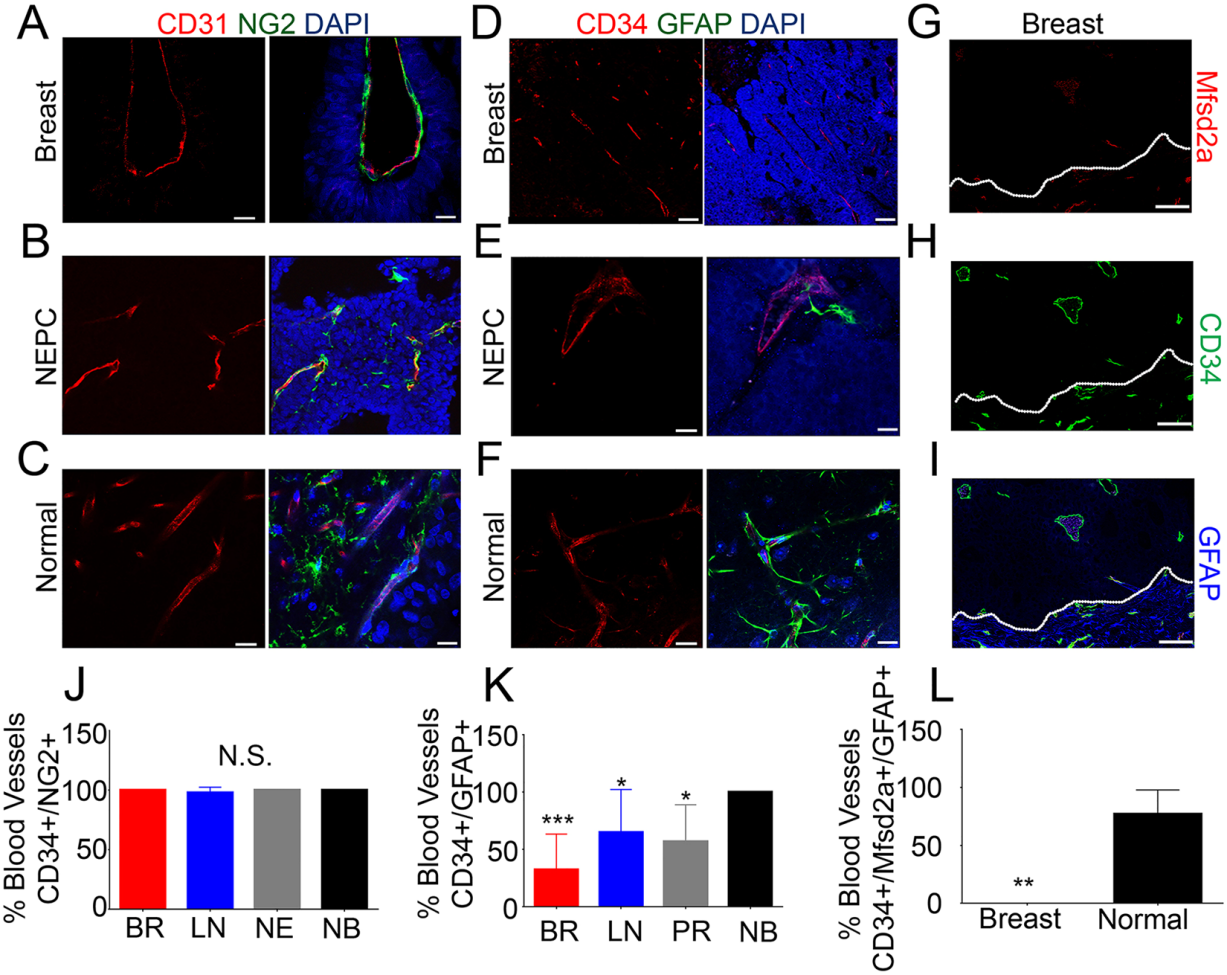
Absence of perivascular astrocytes correlates with reduced Mfsd2a expression in brain metastasis endothelial cells. (**A**–**C**) Double immunofluorescence staining with anti-NG2 and anti-CD31 antibodies in PDX models of breast cancer and NEPC metastases reveals pericytes associated with intratumoral endothelial cells (**A**,**B**) as well as with endothelial cells of the adjacent non-cancerous mouse brain (**C**–**F**) Anti-GFAP and anti-CD34 double labeling in PDX models of metastases reveals a reduction (D), or abnormal association (**E**) of astrocytes with tumor blood vessels as compared to the normal adult mouse brain (**F**–**I**) Triple immunofluorescence labeling with anti-Mfsd2a (**G**), anti-CD34 (**H**) and anti-GFAP (**I**) antibodies in a PDX model of breast cancer brain metastasis reveals lack of Mfsd2a/CD34 double positive blood vessels associated with GFAP-expressing astrocytes compared to the adjacent non-cancerous brain tissue. The dashed line indicates the tumor boundary. (**J**–**L**) Quantitation of CD34^+^ endothelial cells associated with NG2^+^ pericytes (**J**), CD34^+^ endothelial cells associated with GFAP^+^ perivascular astrocytes (**K**), and blood vessels expressing both CD34 and Mfsd2a that are associated with GFAP^+^ perivascular astrocytes (**L**) in PDX models of breast cancer brain metastasis versus adjacent non-cancerous brain tissue, *p < 0.05 and ***p < 0.005. All scale bars are 100 μm.

A previous study reported down-regulation of Mfsd2a in a pericyte-deficient mouse model^11^, suggesting that pericytes are necessary for induction and/or maintenance of Mfsd2a gene expression. Therefore, we investigated whether pericytes were associated with endothelial cells within the metastatic brain tumor vasculature. We found that in metastatic tumors (Fig. 3A,B,J) and in the non-cancerous mouse brain (Fig. 3C), vascular endothelial cells were intimately associated with pericytes expressing the neuron-glia 2 (NG2) protein. Next, we quantified the presence of astrocytes, another abundant cell type in the brain that controls BBB homeostasis. The results indicate a significant reduction of astrocytes throughout the metastatic tumor area, and particularly perivascular astrocytes associated with blood vessels (Fig. 3D,E,K). In contrast, astrocytes were found to fully ensheath blood vessels in non-injected brain regions (Fig. 3F). Occasionally, we observed astrocytes in the vicinity of brain metastasis blood vessels, but these cells often displayed abnormal polarity and defective juxtaposition with tumor endothelial cells (Fig. 3E). Importantly, reduced numbers of perivascular astrocytes strongly correlated with lack of Mfsd2a expression in brain metastasis endothelial cells (Fig. 3G–L). Together, our data support that brain metastasis endothelial cells are intimately associated with pericytes, but show diminished interactions with perivascular astrocytes, and this correlated with reduced expression of Mfsd2a.

**Figure 4:**
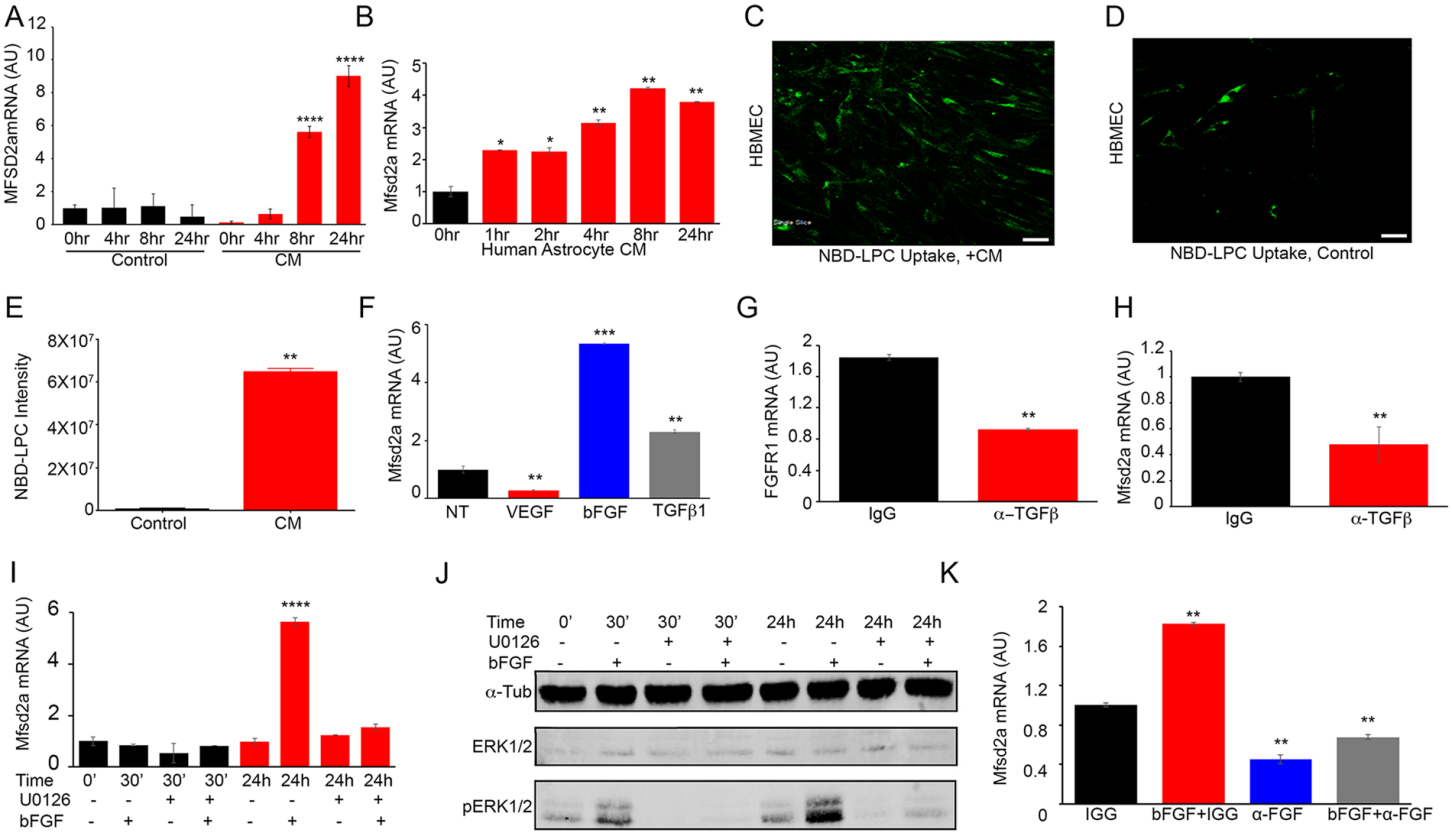
Astrocyte-derived TGFβs and bFGF induce MFSD2A gene expression in cultured brain endothelial cells. (**A**,**B**) Time-dependent induction of MFSD2A mRNA expression in HBMECs exposed to conditioned media taken from mouse astrocytes (A) and human astrocytes (**B**), as revealed by quantitative RT-PCR, *p < 0.01, **p < 0.001. (**C**,**D**) Increased uptake of NBD-LPC by HBMECs pre-treated with astrocyte conditioned media versus non-conditioned control media. Scale bars are 100 μm. (**E**) Quantitation of NBD-LPC fluorescence intensity in HBMECs treated with either control media or brain metastasis spheroid conditioned media, *p < 0.05. (**F**) Quantitation of MFSD2A mRNA expression in HBMECs treated with VEGF-A, TGFβ1, or bFGF, **p < 0.01. (**G**,**H**) Treatment of HBMECs with anti-TGFβ neutralizing antibodies leads to decreased FGFR1 (**G**) and MFSD2A (**H**) gene expression, as revealed by qRT-PCR, **p < 0.01. (**I**–**K**) Inhibition of FGF receptor-dependent signaling via Mek1/2, as assessed by immunoblotting for Erk1/2 phosphorylation status after blocking with U0126 (**I**,**J**) or with anti-FGF neutralizing antibodies leads to reduced MFSD2A gene expression in HBMECs (**K**), **p < 0.01 and ****p < 0.0001. The immunoblot data in (**J**) are cropped images from the same gel.

Since astrocytes were either absent or aberrantly associated with brain metastasis endothelial cells, we hypothesized that cues from astrocytes were important for the normal expression of Mfsd2a. Astrocytes are known to secrete cytokines and growth factors that modulate BBB properties in the brain vascular endothelium^19^. Therefore, we examined the influence of conditioned media taken from primary mouse brain astrocytes or human astrocytes on the expression of Mfsd2a in low passage (<P5) human brain microvascular endothelial cells (HBMECs), which express low endogenous levels of Mfsd2a. Our data showed that both mouse and human astrocytes secrete factors that promote expression of Mfsd2a mRNA in HBMECs (Fig. 4A,B). Mfsd2a is a transporter for DHA when it is conjugated to lysophosphatidylcholine (LPC) in the circulation. Therefore, we examined the effects of astrocyte conditioned media on Mfsd2a-dependent uptake of NBD-LPC, a fluorescent tracer. Our data revealed an increased uptake of NBD-LPC by HBMECs treated with astrocyte-conditioned media, compared to control non-conditioned media (Fig. 4C–E). To identify astroglial-derived factors that positively regulate the expression of Mfsd2a, we treated HBMECs with different cytokines of known effects on endothelial barrier properties, such as VEGF, bFGF/FGF2, and TGFβ1. Levels of Mfsd2a gene expression were measured using qRT-PCR after treatment of HBMECs with each factor. Both bFGF and TGFβ1 induced the expression of MFSD2A in HBMECs, whereas VEGF had an inhibitory effect on MFSD2A gene expression (Fig. 4F).

In support of our findings that TGFβ signaling stimulates Mfsd2a expression in cultured endothelial cells, we analyzed Mfsd2a expression after endothelial cell-specific inhibition of TGFβ signaling in mice. A tamoxifen-inducible transgenic mouse line, PDGFB-CreERT2, that selectively expresses Cre recombinase in vascular endothelial cells^20^ was used to target a conditional type 2 TGFβ receptor allele (Tgfbr2) flanked by loxP sites (Tgfbr2flox/flox)^21^. These mice also harbored a Rosa26-loxSTOPlox-YFP (R26-YFP) fluorescent reporter cassette for identifying Cre-expressing cells based on activation of YFP expression^22^. The type 2 TGFβ receptor (TGFβR2) dimerizes with multiple type 1 receptors and is essential engagement and intracellular signaling. Control (PDGFB-CreERT2; Tgfbr2flox/+; R26-YFP/R26-YFP) and conditional mutant mice (PDGFB-CreERT2; Tgfbr2flox/flox; R26-YFP/R26-YFP) were injected with tamoxifen to activate Cre in the vascular endothelium (Supplemental Figure S7A). Histological analyses revealed obvious disruption of cerebral blood vessel morphologies and permeability after acute genetic deletion of Tgfbr2 (Supplemental Figure S7B,C). Brains of Tgfbr2-knockout and control mice were dissected and cerebral endothelial cells were sorted based on YFP and CD105/Endoglin expression. Quantitative RNA sequencing was performed to identify genes that are differentially expressed in control versus Tgfbr2 knockout endothelial cells (data not shown). Loss of TGFβ receptor signaling led to a significant decrease in Mfsd2a expression in cerebral endothelial cells. Anti-Mfsd2a immunostaining of fixed brain tissue sections also revealed reduced protein expression in Tgfbr2−/− cerebral endothelial cells versus control endothelial cells (Supplemental Figure S8A–C). These data confirm an important role for the TGFβ signaling pathway in the regulation of Mfsd2a expression in vascular endothelial cells.

Tgfbr2 knockout mice additionally showed reduced endothelial cell expression of signaling components of the FGF pathway, including Fgfr1 and Fgfbp1 (Supplemental Figure S8). This was supported by *in vitro* experiments with cultured HBMECs, which demonstrated that expression of both Fgfr1 and Mfsd2a was increased following stimulation with TGFβ1, and suppressed following treatment with an anti-TGFβ neutralizing antibodies (Fig. 4G,H). The FGF pathway is reported to stimulate activation of Erk1/2 in endothelial cells to promote endothelial barrier properties^23^. Treatment of HBMECs with bFGF led to Erk1/2 phosphorylation and increased Mfsd2a gene expression in HBMECs compared to the non-treated control. To further address whether Erk1/2 phosphorylation in response to FGF receptor activation drives Mfsd2a expression, we blocked Mek1/2, which are upstream serine/threonine kinases that phosphorylate and activate Erk1/2. The small molecule Mek1/2 inhibitor, U0126, was added to HBMECs followed by analysis of Erk phosphorylation and Mfsd2a gene expression. Mek1/2 inhibition led to significantly reduced FGF-stimulated Erk1/2 phosphorylation and reduced Mfsd2a expression levels in HBMECs (Fig. 4I,J). In addition, treatment of HBMECs with an anti-FGF2 neutralizing antibody inhibited bFGF-induced Mfsd2a gene expression (Fig. 4K). Collectively, these results reveal a signaling cascade involving TGFβs and bFGF signaling components that positively regulate MFSD2A gene expression in endothelial cells.

**Figure 5:**
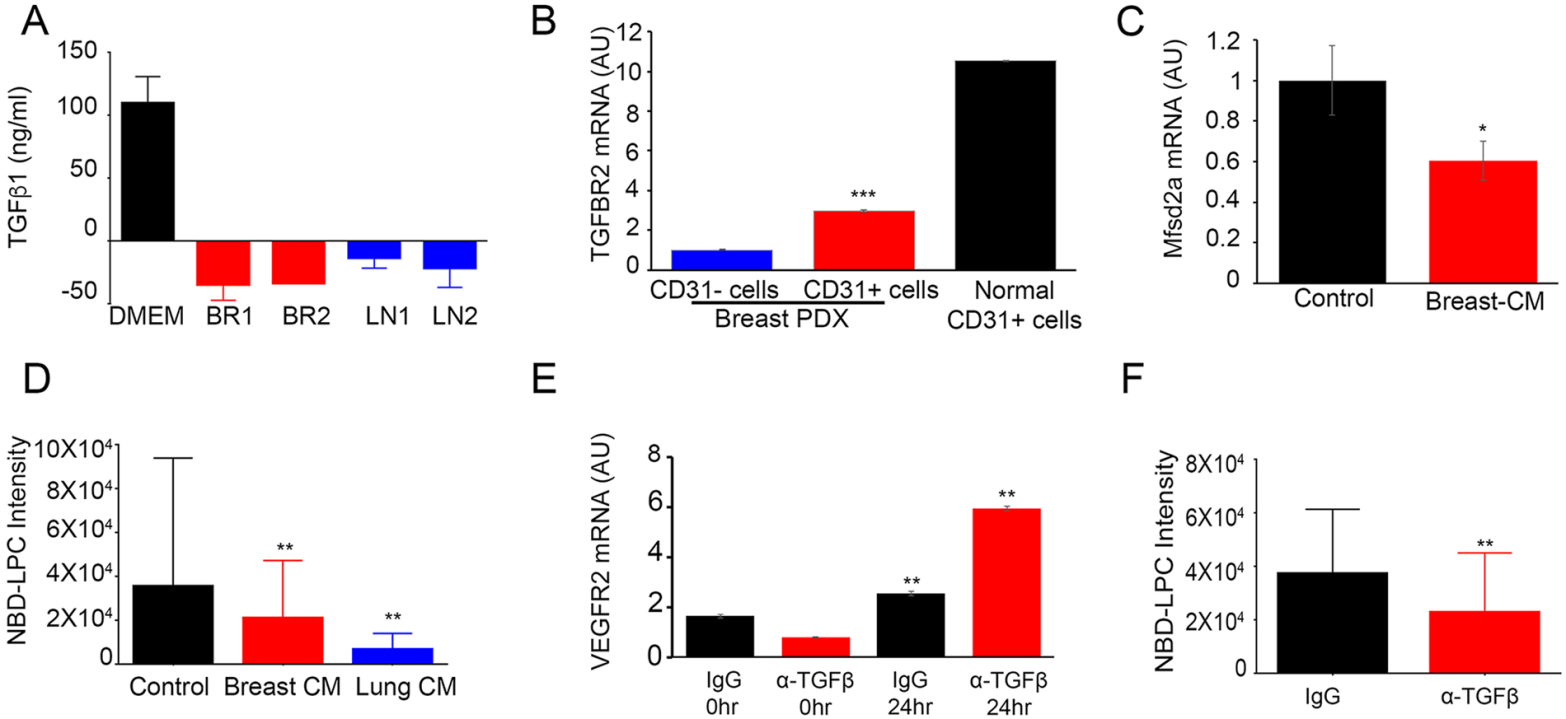
TGFβ1 and bFGF signaling regulates Mfsd2a-dependent endothelial cell barrier functions. (**A**) Conditioned media from primary breast and lung metastatic cells showed significantly reduced TGFβ1 protein levels as measured using a human-specific TGFβ ELISA. (**B**) TGFBR2 gene expression is decreased in CD31^+^ endothelial cells sorted from freshly resected human breast brain metastasis, as compared to CD31^+^ endothelial cells fractionated from adjacent non-cancerous brain tissue, ***p < 0.001. (**C**) HBMECs were treated with conditioned media taken from different primary brain metastasis cells. Note the reduction in MFSD2A gene expression versus non-conditioned control media. (**D**) Reduced uptake of NBD-LPC by HBMECs treated with brain metastasis conditioned media, as compared to non-conditioned media, **p < 0.05 and ****p < 0.001. (**E**) Increased levels of VEGFR2 mRNA expression in HBMECs treated with anti-TGFβ neutralizing antibodies, as compared to treatment with species-matched control IgG, **p < 0.01. (**F**) Decreased NBD-LPC uptake by HBMECs treated with anti-TGFβ neutralizing antibodies as compared to cells treated with IgG control, ***p < 0.001.

Since blood vessels within brain metastases display impaired interactions with astrocytes and reduced astrocyte coverage (Fig. 3), we reasoned that diminished Mfsd2a expression would coincide with reduced levels of astrocyte-derived TGFβs and diminished TGFβ receptor signaling. Indeed, we have reported that latent TGFβ activation by αvβ8 integrin in astrocytes leads to brain vascular pathologies^21,24,25^. Therefore, we quantified levels of TGFβ1 protein in conditioned media harvested from cultured breast cancer and lung cancer brain metastasis cells. The results revealed low levels of TGFβ1 in conditioned media as compared to serum-containing control media (Fig. 5A). Furthermore, we found that TGFBR2 (Fig. 5B) and FGFBP1 (Supplementary Figure S8E) mRNA levels were diminished in CD31^+^ endothelial cell fractions collected from freshly resected human breast brain metastasis, as compared to CD31^+^ endothelial cells fractionated from adjacent non-cancerous brain tissue. Addition of brain metastasis conditioned media exhibited significantly decreased Mfsd2a gene expression in endothelial cells *in vitro* relative to control (Fig. 5C) and also resulted in decreased uptake of fluorescent NBD-LPC (Fig. 5D). Inhibition of TGFβ signaling using neutralizing antibodies led to increased expression of Vegfr2 (Fig. 5E), suggesting that TGFβ signaling suppresses the VEGF-A pathway. Treatment with anti-TGFβ blocking antibodies also resulted in decreased NBD-LPC uptake by HBMECs, further establishing a role for TGFβ signaling in promoting Mfsd2a expression (Fig. 5F).

**Figure 6:**
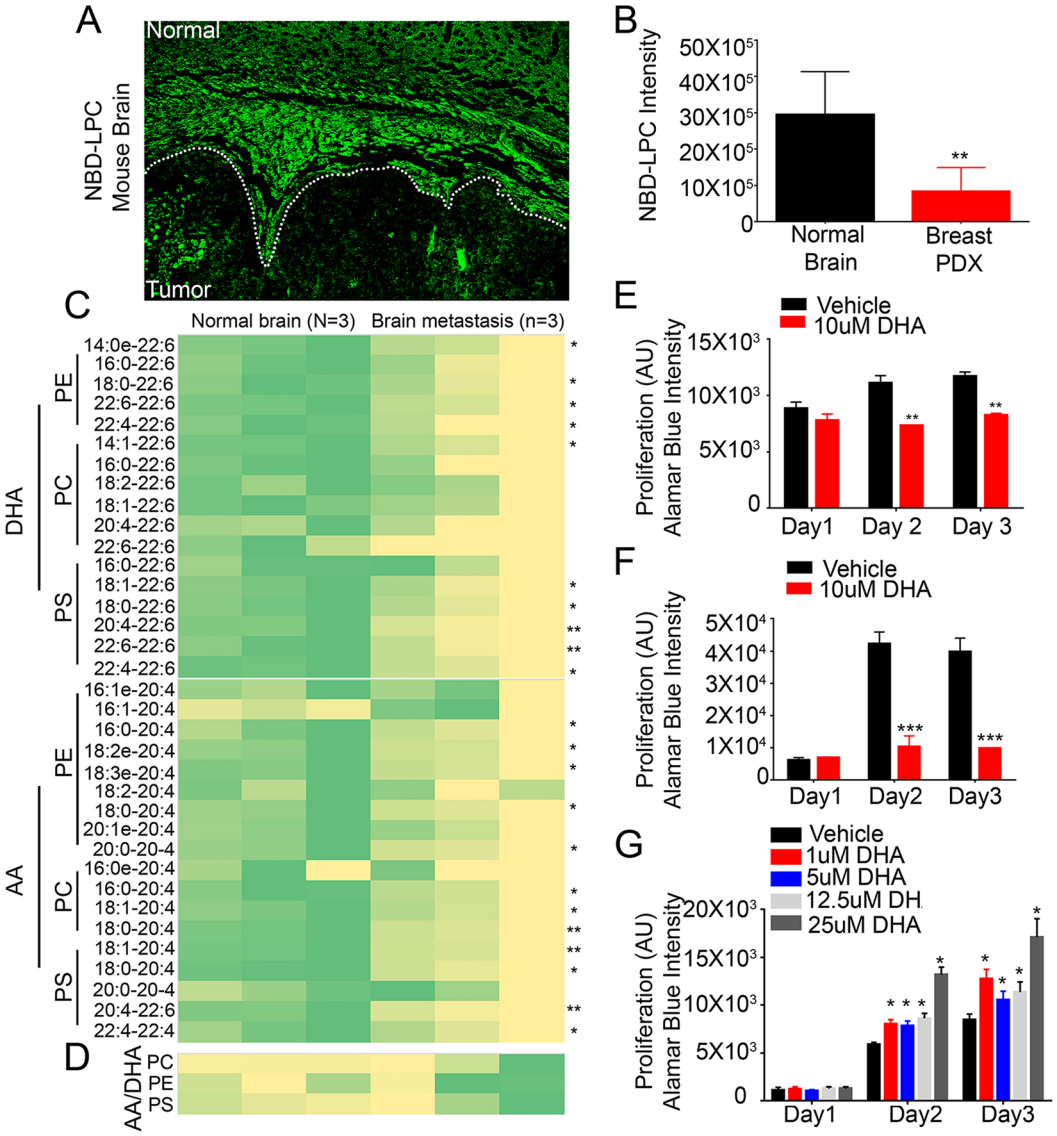
Mfsd2a-mediated DHA transport and lipid metabolism are diminished in PDX models of breast cancer brain metastasis. (**A**) Analysis of NBD-LPC localization in mice harboring breast brain metastasis shows decreased uptake in metastatic tissue (tumor) versus adjacent non-cancerous brain tissue (normal). The dashed line indicates the margin of the brain metastasis. Scale bar is 100 μm. (**B**) Quantitation of NBD-LPC fluorescence intensity in tumor versus normal mouse brain regions after injection of fluorescent NBD-LPC, **p < 0.01. (**C**) Lipidomic heat map showing altered levels of the omega-3 fatty acid DHA and arachidonic acid (AA)-conjugated PE, PC, and PS in tissues from normal mouse brain versus PDX models of breast cancer brain metastasis. (**D**) Lipidomic heat map showing the ratios of total levels of AA to DHA (conjugated to PC, PE and PS) in non-cancerous mouse brain tissue versus breast cancer brain metastasis tissue. (**E**,**F**) Decreased growth and survival of brain metastasis cells derived from breast cancer (**E**) and lung cancer (**F**) after treatment with DHA, **p < 0.05 and ***p < 0.001. (**G**) Treatment with DHA leads to increased proliferation and survival of cultured HBMECs, ***p < 0.001.

Previous studies have shown that Mfsd2a is a transporter for DHA in the brain, with loss of Mfsd2a functions leading to DHA deficiency and related neurological deficits^14,26^. Since our data reveal a significant down-regulation of Mfsd2a expression in the vascular endothelium of brain metastasis, we hypothesized that tumors fail to transport and metabolize DHA, possibly for selective growth and survival advantages. Therefore, we injected tumor-bearing mice with NBD-LPC and measured the fluorescence intensity in the non-cancerous hemisphere and in metastatic tumor tissue. NBD-LPC uptake was significantly decreased in tumor compared to the normal brain, as indicated by reduced intratumoral fluorescence (Fig. 6A,B). To test whether levels of DHA were affected as a result of Mfsd2a down-regulation in the tumor endothelium, we also performed ‘lipidomic’ analyses using tumor tissue dissected from PDX models of breast cancer brain metastasis or matched tissue dissected from the non-injected brain hemisphere. Levels of phosphatidylethanolamine (PE), phosphatidylcholine (PC) and phosphatidylserine (PS) conjugated to DHA were quantified by mass spectrometry. The data revealed decreased levels of DHA-conjugated PE, PC and PS in the brain metastasis tumor area versus the normal brain (Fig. 6C and Supplemental Figure S9). Other studies have reported increased levels of omega-6 fatty acids, especially arachidonic acid (AA) in response to reduced levels of DHA^26^. Therefore, we also quantified levels of AA-containing PE, PC and PS in brain metastases tissue and in tissue from the non-injected half of the brain. Interestingly, levels of AA-conjugated PE, PC and PS species were reduced in metastatic tumor tissue versus the normal brain, suggesting an overall down-regulation of essential fatty acid metabolism in brain metastases (Fig. 6C). Even though levels of both DHA and AA conjugated species were reduced in tumor samples, our data showed an overall increase in the ratio of AA (omega 6) to DHA (omega 3) conjugated PE, PC and PS in the brain metastasis compared to the non-injected hemisphere (Fig. 6D and Supplemental Figure S9). Lastly, to evaluate a direct effect of DHA on metastases, we treated cultured primary lung and breast brain metastasis spheroids with DHA. We detected decreased growth and survival of tumor cells treated with DHA compared to the vehicle control (Fig. 6E,F). In contrast, DHA treatment promoted the growth and survival of HBMECs (Fig. 6G). Collectively, these data reveal that loss of Mfsd2a in metastatic tumor endothelial cells leads to decreased uptake of essential fatty acids, especially DHA, which may promote tumor growth and survival in the brain microenvironment.

## Discussion

The BBB regulates the passage of ions, proteins, and cells between the circulation and the brain to control proper development and physiology. These selective permeability properties also limit the penetration of therapeutics to treat brain pathologies. Indeed, most drugs and biologics fail to exit the circulation and reach targets within the neural parenchyma due to exclusion by the BBB^8^. Drug resistance in brain metastases is classically attributed the inability of agents to reach the tumor microenvironment due to exclusion by the BBB. However, brain metastases demonstrate contrast-enhancement on magnetic resonance and computed tomography scans indicate some degree of BBB disruption^27^. Physiological control of the BBB involves endothelial cell-intrinsic pathways as well as local cues that regulate the formation of endothelial tight junctions, activities of transporter proteins, and/or transcellular endocytosis^28^. Our analysis of fluorescent tracers and circulating biomarkers in the PDX mouse models and patient samples reveal heterogeneous leakage in different tumor regions. Some blood vessels displayed apparently normal BBB functions, whereas others were highly permeable likely due to loss of Mfsd2a and deregulation of endothelial transcytosis. A ‘blood-tumor barrier’ may also affect heterogeneity of vascular leakage, with closely juxtaposed tumor cells inhibiting the passage of circulating factors across the endothelium. This could explain the expression of adherens and tight junction proteins such as VE-cadherin and claudin 3 in some metastatic tumor cells. Indeed, in breast cancer brain metastases we detect blood-vessel-like channels lacking CD31^+^ endothelial cells that are likely formed by tumor cells. Similarly, expression of the glucose transporter Glut1 in metastatic tumor cells may be an additional regulatory mechanism to control intratumoral glucose levels and metabolism. It is enticing to speculate that down-regulation of Mfsd2a may be a strategy utilized by tumor cells to specifically block DHA transport and generally disrupt the BBB via activation of transcytosis events. A secondary blood-tumor barrier would compensate for loss of the BBB to inhibit unwanted components, such as growth-inhibitory cells and proteins, from exiting the circulation and entering the cancer microenvironment.

Pericytes are necessary for normal formation and homeostasis of the BBB^29,30^. However, our data shows that tumor endothelial cells exhibit decreased Mfsd2a expression despite association with pericytes. This is in contrast to a prior report showing that in the Pdgfb^ret/ret^ mouse model, which has reduced numbers of brain pericytes, there is reduced expression of Mfsd2a protein in cerebral endothelial cells^11^. We propose that low Mfsd2a levels in mice deficient in pericytes may be due to a disruption of the normal juxtaposition between astrocytes and cerebral blood vessels, leading to diminished astrocyte-induced levels of Mfsd2a gene expression. Indeed, we provide evidence that Mfsd2a expression in cultured cerebral endothelial cells is induced by astrocyte-derived TGFβ1. In addition, PDX models exhibited reduced numbers of perivascular GFAP-expressing astrocytes and reduced expression of TGFβ1, which correlated with low Mfsd2a expression and increased fluorescent tracer extravasation across the BBB. Therefore, our results demonstrate a critical role for the TGFβ pathway in Mfsd2a-dependent DHA transport and metabolism. TGFβ’s are secreted as latent ECM-bound complexes that are activated through various mechanisms, including adhesion by integrin receptors^31^. αvβ8 integrin expressed in glial cells binds to latent-TGFβ1/3 in the ECM and mediates TGFβ activation and signaling in endothelial cell sprouting and permeability^32^. Selective deletion of αv or β8 integrin gene expression during development leads to angiogenesis pathologies and intracerebral hemorrhage. Similar phenotypes occur upon inhibition of TGFβ signaling, either through Tgfbr2 or Alk5 gene deletion, during mouse development^24,25^. Inhibition of the TGFβ signaling pathway in adult mice by delivering soluble forms of endoglin leads to acute defects in vascular permeability^33^. Interestingly, by four to six post-natal weeks of age, 100% of surviving αv and β8 integrin mutant mice have smaller brains and display neurological phenotypes, including sporadic seizures and a rigid gait^34^. These neurological abnormalities become progressively more severe, and by four months mutant mice display ataxia associated with neurodegeneration in white matter tracts of the cerebellum and spinal cord. The phenotypes in itgav and itgb8 mutant mice are similar to motor deficits that develop in patients with DHA deficiency. Based on RNA sequencing data linking TGFβ signaling to Mfsd2a expression and function, we propose that integrin mutant phenotypes are due, in part, to DHA deficiency caused by diminished TGFβ signaling and reduced expression of Mfsd2a in endothelial cells. It will interesting to determine whether adult mice genetically null for αv or β8 integrins express reduced levels of Mfsd2a in the cerebral vascualture, and whether this is associated with altered DHA transport and lipid metabolism.

FGF receptor signaling has been reported to control endothelial cell permeability by modulating adherens junction integrity^35^. One recent report showed synergistic interactions between TGFβ and FGF pathways in endothelial cells outside of the brain^36^. Interestingly, we found that ablation of Tgfbr2 results in a significant loss of Mfsd2a, Fgfbp1, and Fgfr1 gene expression in the brain endothelium. Fgfr3 is also expressed in the vascular endothelium and genetic studies in mice have shown that Fgfr1 and Fgfr3 are both required for physiological angiogenesis and cardiovascular development^37^ via activation of the glycolytic enzyme hexokinase 2. Acute inhibition of FGF signaling in endothelial cells has also been reported to be an effective anti-angiogenic strategy in mouse models of cancer^38^. In cerebral endothelial cells we have shown that TGFβ1 is an upstream regulator of FGF receptor gene expression and signaling, with these pathways cooperatively promoting expression of Mfsd2a. Hence, we predict that antagonism of the FGF pathway, while potentially effective as an anti-angiogenic strategy for cancer and other pathologies, may lead to unexpected alterations in the BBB and defects in lipid metabolism due to inhibition of Mfsd2a.

Omega-3 and omega-6 polyunsaturated fatty acids (PUFAs) contribute to the development and physiology of multiple organs, including the brain^39^. The two major PUFAs present in the brain are DHA (omega-3) and AA (omega-6). DHA is not synthesized by the body, but is obtained exclusively via dietary sources. DHA is incorporated into phospholipids in cell membranes, phospholipase-mediated cleavage generating various lipid metabolites^40^. In general, DHA and its metabolites such as resolvins, neuroprotectins, and maresins have systemic anti-inflammatory and tumor suppressive effects, whereas AA and its metabolites (leukotrienes, prostaglandins, and thromboxane) have pro-inflammatory functions and tumor-promoting effects^41^. Mfsd2a transports DHA that is conjugated to LPC^14,26^. Consistent with these data, we have found a decrease in proliferation of tumor cells isolated from breast and lung brain metastasis after treatment with DHA. Other than omega-3 fatty acids, the brain contains high levels of omega-6 fatty acids, which possess pro-inflammatory and tumor promoting properties. Our data reveal a decrease in overall levels of AA conjugated PC, PE and PS in mouse models of breast cancer brain metastasis. This is in agreement with other studies which report an increased ratio of omega-6 to omega-3 fatty acids in pathological conditions including cancer^42^.

DHA and its lipid metabolites have established roles as anti-cancer agents. Use of DHA as an adjuvant increased efficacy of treatment in cancer patients^43^. Multiple studies have reported its anti-tumor activity^44^. In addition, transgenic MMTV-Her2/Neu mice show a 30% reduction in mammary tumor incidence when fed a diet rich in omega-3 PUFAs^45^. In humans, dietary fish oil supplementation is associated with decreased risk for glioma^46^ and breast cancer^47^. Cancer patients with DHA-rich diets (>1:1 ratio of omega-3:omega-6 PUFAs) also display more favorable responses to chemotherapies^48^. In addition, a DHA-rich diet is reported to reduce neurocognitive deficits in metastatic breast cancer patients receiving whole brain irradiation^49,50^. The mechanisms underlying how DHA and its metabolites elicit these phenomena and how tumor cells may alter their microenvironment to circumvent those inhibitory effects remain unknown. Based on our data, we propose that an omega-3 PUFA-rich diet suppresses metastatic malignancy by increasing levels of DHA in the brain, perhaps by inhibiting early seeding and growth of neoplastic cells in the brain microenvironment prior to their eventual inhibition of Mfsd2a and DHA transport. Therefore, restoring DHA levels in brain metastases may be a potential “chink in the armor” that can be exploited for therapeutic intervention to diminish cancer cell growth and survival.

## Methods

### Ethics statement

Approval for the use of human specimens was obtained from the Institutional Review Board (IRB) at the University of Texas MD Anderson Cancer Center. The IRB waived the requirement for informed consent for previously collected residual tissues from surgical procedures stripped of unique patient identifiers according to the Declaration of Helsinki guidelines.

### Experimental mice

All mouse experiments were reviewed and approved prior to animal use under the guidance of the Institutional Animal Care and Use Committee (IACUC) and the MD Anderson Subcommittee on Animal Studies, both AAALAC accredited institutions. Male NOD-SCID mice (4–8 weeks old) were purchased from Jackson Laboratories and used for all experiments involving intracranial injections of metastatic tumor cells. The Tgfbr2flox/flox^22^, PDGFB-CreERT2^20^, and Rosa26-loxSTOPlox-YFP^22^ mouse strains (on a C57Bl6/129S4 genetic background)have been described elsewhere. Pups (P1-P3) were injected in the stomach with 50 μl tamoxifen (1 mg/ml) (Fisher Scientific, #54965–24–1) prepared in sterile corn oil. Mice were sacrificed at P7 for cerebral endothelial cell fractionation or immunohistochemical analyses. Genotypes were confirmed using PCR and genomic DNA isolated from tail or ear snips.

### Generation of primary cell cultures and PDX mouse models of brain metastasis

Freshly resected human brain metastasis originating from different primary cancer types (breast, lung, melanoma, neuroendocrine prostate, and others) were obtained normally within two hours after surgery via a clinical protocol approved by the IRB at the University of Texas MD Anderson Cancer Center. Genetic, histologic and biomarker analysis of metastatic tumors were performed to confirm the primary cancer of origin. The brain metastasis samples were dissociated using a brain tissue dissociation kit (Miltenyi Biotec # 130-095-942). Single cells were injected into NOD-SCID mice using intracranial guided screw method or cultured *in vitro* in DMEM F12 (Mediatech, Manassas, VA, USA) supplemented with 20 ng/ml EGF and bFGF (Gibco, Hilden, Germany), B27 supplement (Life Technologies, Carlsbad, CA, USA) and one unit per ml penicillin-streptomycin (Gibco). For analysis of tumor growth and progression, mice were euthanized using CO_2_ followed by dissection of the brain to obtain tumor tissue followed by dissociation and secondary implantation into NOD SCID mice. To obtain fixed tumor tissue, tumor-bearing mice were deeply anaesthetized followed by cardiac perfusion with 4% paraformaldehyde (PFA)/phosphate-buffered saline (PBS). The brain was removed and fixed in 4% PFA/PBS overnight at 4 °C prior to tissue processing and experimental analysis.

### Microscopy and antibodies

Images were acquired using an Olympus FLUOVIEW FV1000 confocal microscope. Fluorescence intensity was measured using ImageJ or Olympus FLUOVIEW software. The Pearson’s coefficient for co-localization was measured using Olympus FLUOVIEW software. Commercially available antibodies used for immunoblotting and immunostaining of tissue sections include rabbit anti-MFSD2a (Cell Signaling Technology, Beverly, MA), mouse anti-E-cadherin (cat#610181, BD, Biosciences, San Diego, CA, USA), rabbit anti-NG2 (Millipore), rabbit anti-GFAP (cat#Z0334, DAKO, Glostrup, Denmark), chicken anti-Nestin (cat#CH23001, Neuromics, Minneapolis, MN, USA), rat anti-CD31 (cat#553370, BD Biosciences, San Diego, CA, USA), rat anti-CD34 (GTX28158, GeneTex, Irvine, CA, USA), mouse-anti-Erk1/2 (cat#9107 S, Cell Signaling Technology), and rabbit-anti-pErk1/2 (cat#4370 s, Cell Signaling Technology). Secondary antibodies include anti-rabbit IgG Alexa Fluor-594/488, 1:500 (cat#711-585-152/cat#711-545-152, Jackson labs), anti-rat IgG Alexa Fluor-594, 1:500 (cat#112-585-167, Jackson labs), anti-mouse IgG Dylight-405, 1:500 (Jackson labs), and anti-rabbit IgG DyLight-405,1:500 (cat#711-475-152, Jackson labs). Secondary antibodies used for immunoblotting were IRDye 800CW anti-rabbit, 1:10,000 (LICOR) and IRDye 680RD anti-mouse, 1:10,000 (LICOR).

### Cell culture studies

Primary HBMECs were purchased from ScienCell™ Research Laboratories and used at low passage number (<P5). Cells were cultured in ECM medium (ScienCell™1001) in 5% FBS and ECGS. Astrocytes were isolated from the brains of mouse pups at post-natal day 3 and cultured in low glucose DMEM medium from ThermoFisher (sh3002101). Normal Human Astrocytes were purchased from Lonza (CC-2565) and cultured in ABM basal media with supplemental growth factors (Lonza CC-4123). HBMECs were plated at 70% confluence and cultured in conditioned media from both astrocytes and tumor cells were collected at 72 hours for treatment. The growth factors and concentrations used for endothelial cell treatment were 0.1 ng/ml for TGFβ1 (R&D Systems, 240-B-002), 100 ng/ml for bFGF (Gibco), and 100 ng/ml for VEGFA (Biolegend 583702). The neutralizing antibodies used are anti-TGFβ (1–4 μg/ml, R&D Systems, MAB1835) and anti-bFGF (16 μg/ml, R&D Systems, AF-233). U0126 inhibitor (20 μM, Tocris) was used to inhibit Mek1/2-mediated phosphorylation of Erk in HBMEC cells. Concentrated lentivirus for pLOC-MFSD2a (Clone ID-PLOHS_100073371) and control pLOC were obtained from the MD Anderson Open Reading Frame core facility. HBMECs were infected at MOI 5. Cells overexpressing MFSD2a were lysed and levels of mRNA expression were measured by real time PCR. For the TGFβ1 ELISAs, 250,000 metastatic cells were plated in a 6 well plate and conditioned media was collected at 72 hours for treatment. The levels of TGFβ1 were measured using human TGFβ1 ELISA kits (R&D Systems) according the manufacturer’s protocol.

### NBD-LPC uptake assays

Tumor-bearing mice were injected intraperitoneally with 300 µg of NBD-LPC (Avanti-Polar #810128 P) diluted in PBS (total volume per mouse was 200 µl). Two hours later, mice were sacrificed and cardiac-perfused with 4% PFA/PBS. The brain was excised and fixed overnight at 4 °C. On the next day, the brain was cut coronally at the injection site and embedded in 4% agarose followed by sectioning with a vibratome. The sections were mounted and NBD-LPC signal was detected using an Olympus confocal microscope. The fluorescence intensity of the signal between the tumor-inject hemisphere and normal non-injected half of the brain was measured using Olympus software. For *in vitro* assays, cells were plated on collagen coated glass coverslips and allowed to adhere. This was followed by overnight serum starvation. Cells were subsequently treated with different ligands/conditioned media for 24 hours. The cells were washed with PBS followed by incubation with 10 µg NBD-LPC diluted in media for 30 minutes. Cells were then washed with PBS and mounted on glass slides. Confocal microscopy was used to detect the NBD-LPC signal and fluorescence intensity was measured using Olympus software.

### Lipidomic analysis

 Tumor tissue and healthy non-injected brain were flash frozen in liquid nitrogen. Frozen tissue samples were shipped to Avanti polar lipids (Alabaster, Alabama, USA) to quantify the DHA (docosahexanoic acid), AA (arachidonic acid) conjugated PC (phosphatidylcholine), PS (Phosphatidylserine) and PE (Phosphatidylethanolamine). For lipid extraction and measurement of each species, tissue samples were weighed and homogenized with HPLC grade water. Lipids were extracted in 1:1 dicholoromethane:methanol and dried at 37 °C using a rotary vacuum evaporator. The residue was dissolved in 1 ml of 1:1 dichloromethane:methanol for further analysis and detection of lipid species by LC/MS.

### Reverse transcription, real time PCR, and overexpression

An RNAeasy kit (Qiagen, Hilden, Germany) was used to extract total RNA. Reverse transcription was performed to obtain cDNA using SuperScript VILO Master Mix (ThermoFisher) followed by quoin of MFSD2A fold-change by RT-PCR using the following primers: MFSD2a:F-CTCCTGGCCATCATGCTCTC, R-GGCCACCAAGATGAGAAA; β-actin: F-TCCCTGGAGAAGAGCTACGA R:AGCACTGTGTTGGCGTACAG; GAPDH: F-CAGAACATCATCCCTGCCTC, R-TGGCAGGTTTTTCTAGACGG; FGFR1: F-AACCTGACCACAGAATTGGAGGC, R: ATGCTGCCGTACTCATTCTCCACA; FGFBP1:F-CTTCACAGCAAAGTGGTCTCA, R-GACACAGGAAAATTCATGGTCCA; TGFBR2: F-GTAGCTCTGATGAGTGCAATGAC,R-CAGATATGGCAACTCCCAGTG.

### BBB permeability assays

Evans blue (EB, 4%) in PBS was injected into tumor bearing mice via the tail vein. Cardiac perfusion using 4% PFA/PBS was performed two hours after injection and the brain was excised and fixed overnight at 4 °C. The following fay, brains were cut into two halves at the injection site and the tissue was embedded in 4% agarose. 100 µm thick sections were generated from the agarose embedded tissue using a vibratome. The sections were permeabilized in 0.2% Triton X-100 in PBS for 15 minutes followed by three washes with PBS. Sections were blocked with 10% serum matching the secondary host for one hour at room temperature. Primary antibody (m-anti-CD31) incubation was performed overnight at 4 °C. Next day, the sections were washed with PBS-T (Triton 0.01%) four times each for 30 minutes. Secondary antibody was added to the sections and incubated at room temperature for 2 hours, followed by four 30-minute washes. Finally the sections were mounted on glass slides using Vectashield with DAPI (Vector Labs, Burlingame, CA, USA). The EB signal and CD31 immunofluorescence were imaged using a confocal microscope. The BBB leakage was determined by measuring the co-localization of CD31-expressing blood vessels and EB fluorescent signal. Pearson’s coefficient was calculated, for linear correlation indicating co-localization of CD31 and EB fluorescence signal between the non-injected half of the brain and tumor area using the Olympus software.

Cardiac perfusion of deeply anaesthetized tumor bearing mice was preformed using 7.5 ml of 1 mg/ml of EZ-link™ Sulfo-NHS-Biotin (Thermo Scientific #21217) in PBS. This was followed by a 4% PFA/PBS perfusion and excision of brain and overnight fixation at 4 °C. Next day, the brains were cut coronally at the injection site, fixed and embedded in paraffin. Formalin fixed/paraffin embedded slides were obtained from tumor-bearing mice that had been perfused with sulfo-NHS-biotin. Immunostaining was performed for CD34 and biotin signal was detected by using streptavidin-488. The BBB leakage was determined by measuring the co-localization for CD34 and biotin-streptavidin signal. Pearson’s coefficient was calculated, for co-localization of CD34 and biotin-streptavidin fluorescence signal between the non-injected half of the brain and the tumor-containing brain region, using the Olympus software.

### Whole transcriptome analysis

Control and Tgfbr2 knockout endothelial cell pellets were snap frozen and total RNA was isolated using in-house methods at Expression Analysis/Q2 solutions. After RNA quality was validated, six samples with a RIN of ≥7 (n = 3 control and n = 3 knockout) were selected. An average of ~75 million paired-end reads were generated for each of the six samples. Sequencing quality evaluation, alignment of short sequenced reads, and expression calling using reads per kilobase per million reads (RPKM) values were performed by utilizing the Pipeline for RNA Sequencing Data Analysis (PRADA). The expression values of a total of 20,009 protein-coding genes (Ensembl reference transcriptome version 64) were calculated per the RPKM value of their longest transcript. The signal-to-noise metric was used to calculate the gene expression differences between cell samples.

### Statistical analysis

Statistical differences were calculated by using unpaired student’s t-test and two-way ANOVA; a p-Value of <0.05 was considered significantly different. All statistical tests were performed on Graph Pad Prism 6.

### Data availability statement

All data generated or analyzed during this study will be included in this published article or its Supplementary Data files. The authors will make materials and associated protocols promptly available to readers without undue qualifications in material transfer agreements.

### Ethical approval and informed consent

All mouse experiments were approved prior to animal use in accordance with the Institutional Animal Care and Use Committee (IACUC) and the MD Anderson Subcommittee on Animal Studies. Approval for the use of human specimens was obtained in accordance with the Institutional Review Board (IRB) at the University of Texas MD Anderson Cancer Center. The IRB waived the requirement for informed consent for previously collected residual tissues from surgical procedures stripped of unique patient identifiers. Otherwise, informed consent was obtained prior to surgery and collection of patient tumor specimens.

## Electronic supplementary material

Supplemental Figures and Legends
