# Supplementary material for: Metastatic Brain Tumors Disrupt the Blood-Brain Barrier and Alter Lipid Metabolism by Inhibiting Expression of the Endothelial Cell Fatty Acid Transporter Mfsd2a
Source: Sci Rep. 2018 May 29;8:8267. doi: 10.1038/s41598-018-26636-6 (PMC5974340; doi:10.1038/s41598-018-26636-6)

Supplementary Information

Title: Metastatic Brain Tumors Disrupt the Blood-Brain Barrier and Alter Lipid Metabolism by Inhibiting TGFβ-Mediated Expression of the Endothelial Cell Fatty Acid Transporter Mfsd2a

Authors: Shweta Tiwary, John E. Morales, Sam C. Kwiatkowski, Frederick F. Lang, Ganesh Rao, and Joseph H. McCarty

Supplemental Figure Legends

Supplemental Figure S1. Primary human brain metastatic cells grow as neurosphere-like spheroids in vitro. Tumor cells were cultured from freshly resected human brain metastases derived from breast cancer (top) and NEPC (bottom) in serum-free media containing FGF and EGF. Note that brain metastatic cells grow as free-floating spheroids and express the epithelial markers E-cadherin (green) and β-catenin (red). Scale bars are 100 μm.

Supplemental Figure S2. Histological analysis of human brain metastasis growth features in the NOD-SCID mouse brain. (A-D); Tumor cells were dissociated from freshly resected human brain metastases and injected intracranially into NOD-SCID mice. In comparison to the normal mouse brain (A), note that cultured metastatic tumor cells originating from breast cancer (B), NEPC (C), and lung cancer (D) form large epithelial-like tumors in the brain as revealed by H&E staining. Scale bars are 100 μm.

Supplemental Figure S3. Immunofluorescence analysis of human brain metastasis growth features in the NOD-SCID mouse brain. (A, B); Dissociated NEPC brain metastasis cells were injected into the brains of NOD-SCID mice. Note that tumor cells form epithelial-like tumors in the brain, as revealed by anti-E-cadherin staining (green). Tumors are well vascularized based on CD31/PECAM expression (red), display non-invasive growth properties, and have clearly defined boundaries. Growth patterns observed in PDX models of brain metastasis are very similar to what is detected in primary patient samples. Scale bars are 100 μm.

Supplemental Figure S4. E-cadherin protein is expressed in human brain metastasis samples. (A, B); Anti-E-cadherin immunofluorescence staining of formalin fixed and paraffin embedded human breast (A) and lung (B) brain metastasis specimens. Note the localization of E-cadherin protein is primarily in the membrane of tumor cells. Scale bars are 100 μm.

Supplemental Figure S5. Heterogeneous disruption of the BBB in fixed human breast cancer brain metastasis samples. (A-F); Formalin fixed paraffin embedded samples from normal human brain (A, B) or two different human breast cancer brain metastases (C-F) were immunostained with H&E (A, C, E) or antibodies recognizing human IgG (B, D, F). Note that human IgG does not normally cross the intact BBB (red arrows, B), whereas in metastatic tumors there is heterogeneous extravasation of human IgG protein. Some intratumoral blood vessels display an intact BBB (red arrows, D), but other blood vessels have more permeable properties as evidenced by IgG leakage into the tumor microenvironment (F). Scale bars are 100 μm.

Supplemental Figure S6. Metastatic tumor cells express BBB adherens junction and tight junction proteins. (A, B); Anti-VE-cadherin antibody labeling reveals VE-cadherin protein expression in tumors cells of breast cancer brain metastasis (A) and lung cancer brain metastasis (B). **(C, D);** Expression of Glut 1 in tumor cells in PDX models of breast cancer brain metastasis (C) and lung cancer brain metastasis (D), as revealed by immunohistochemical labeling. **(E);** Analysis of Claudin 3 protein expression in PDX models of breast cancer brain metastasis (top) and NEPC brain metastasis (bottom). Note that Claudin 3 protein is expressed mainly in metastatic tumor cells, and to a lesser degree in vascular endothelial cells. Scale bars are 100 μm.

Supplemental Figure S7. Tamoxifen activates Cre and induces Tgfbr2 gene deletion in vascular endothelial cells. (A); Tamoxifen activation of PDGFB-CreERT2 leads to endothelial-specific expression in cerebral endothelial cells, as revealed with a Rosa26-loxSTOPlox-YFP reporter strain. P7 brain sections were immunofluorescently labeled with anti-CD31 and anti-GFP antibodies, revealing co-localization in brain endothelial cells. **(B);** Brain sections from control and mutant (Tgfbr2 ECKO) P7 mice were analyzed by immunostaining with anti-CD31 and anti-NG2 antibodies, revealing BBB disruption and vascular leakage in mutant mice. Scale bars are 100 μm.

Supplemental Figure S8. Quantitation of TGFβ-dependent Mfsd2a gene expression in the mouse brain. (A, B); Analysis of Mfsd2a (left) and CD31 (right) protein expression in the brain of PDGFB-CreERT2;R26-YFP;Tgfbr2f/+ (control, A) and PDGFB-CreERT2;R26-YFP;Tgfbr2f/f (TGFBR2 ECKO, C) mice at post-natal day 7 (P7), showing a decrease in Mfsd2a protein levels in endothelial cells. Mice had been injected with tamoxifen at P1-P3. **(C);** Quantitation of anti-Mfsd2a immunofluorescence intensity in P7 control and TGFBR2 KO mice, *$p<0.05$. **(D);** Decreased relative expression of FGFBP1, FGFR1 and Mfsd2a, as measured by quantitative RNA sequencing from endothelial cells sorted from control and KO P7 mouse brain, **$p<0.009$, ***$p<0.0001$. **(E);** Quantitation of relative expression levels of FGFBP1 in CD31-expressing endothelial cells fractionated from freshly resected breast cancer brain metastasis tissue versus non-cancerous human brain tissue, **$p<0.01$. Scale bars are 100 μm.

Tiwary et al., Supp Figure S1

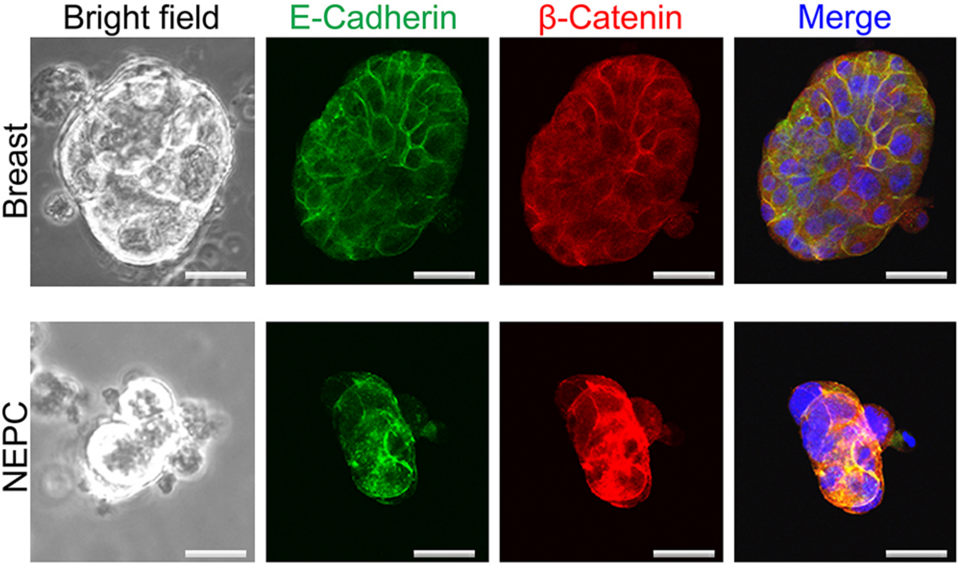

Tiwary et al., Supp Figure S2

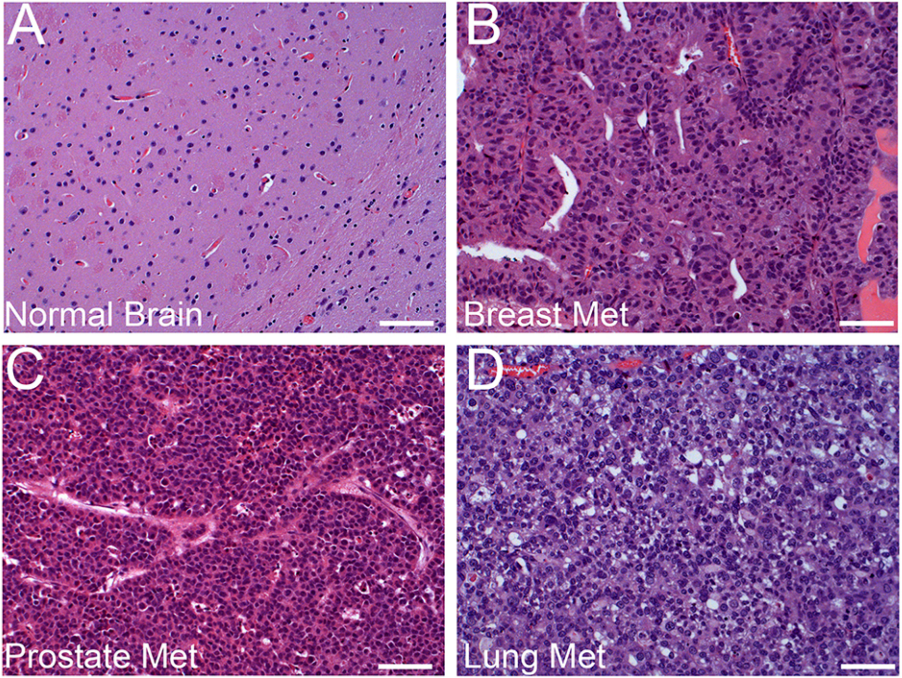

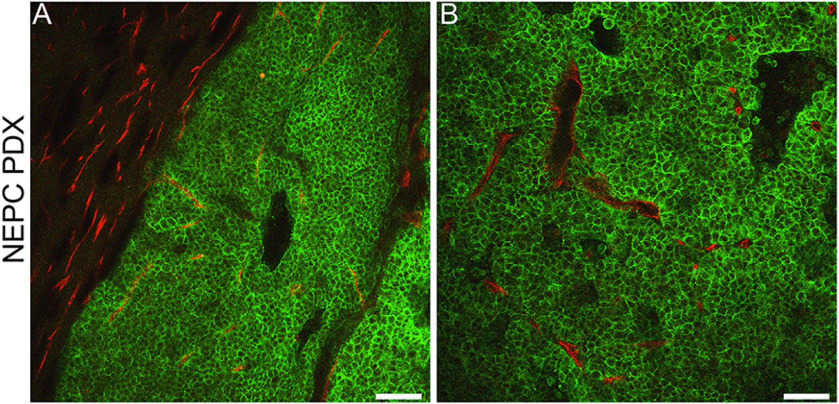

E-Cadherin CD31

A

Human breast brain metastasis

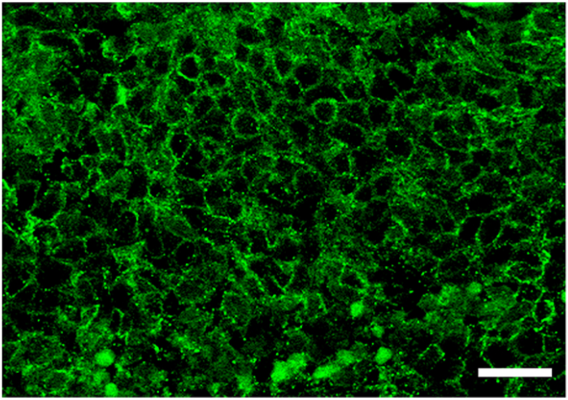

B

Human Lung brain metastasis

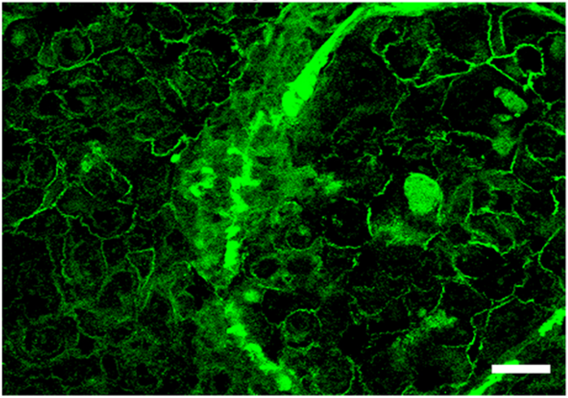

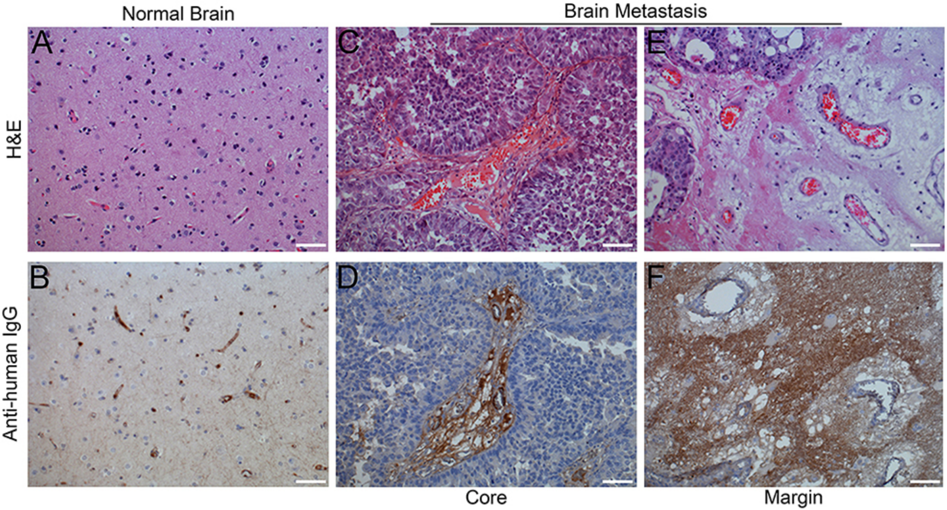
Normal Brain
Brain Metastasis
A
C
E
B
D
F
H&E
Anti-human IgG
Core
Margin

Tiwary et al., Supp Figure S6

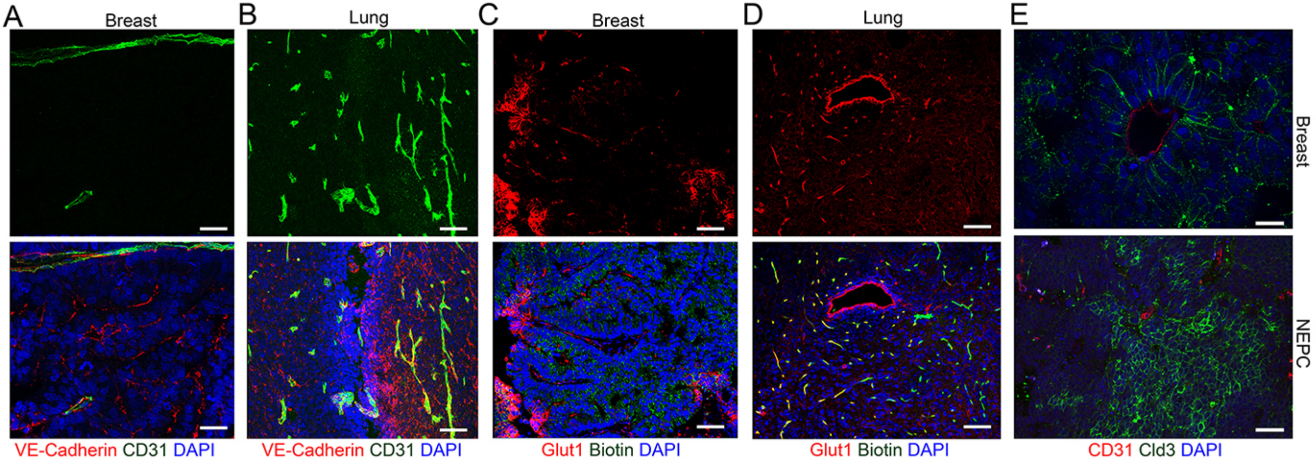

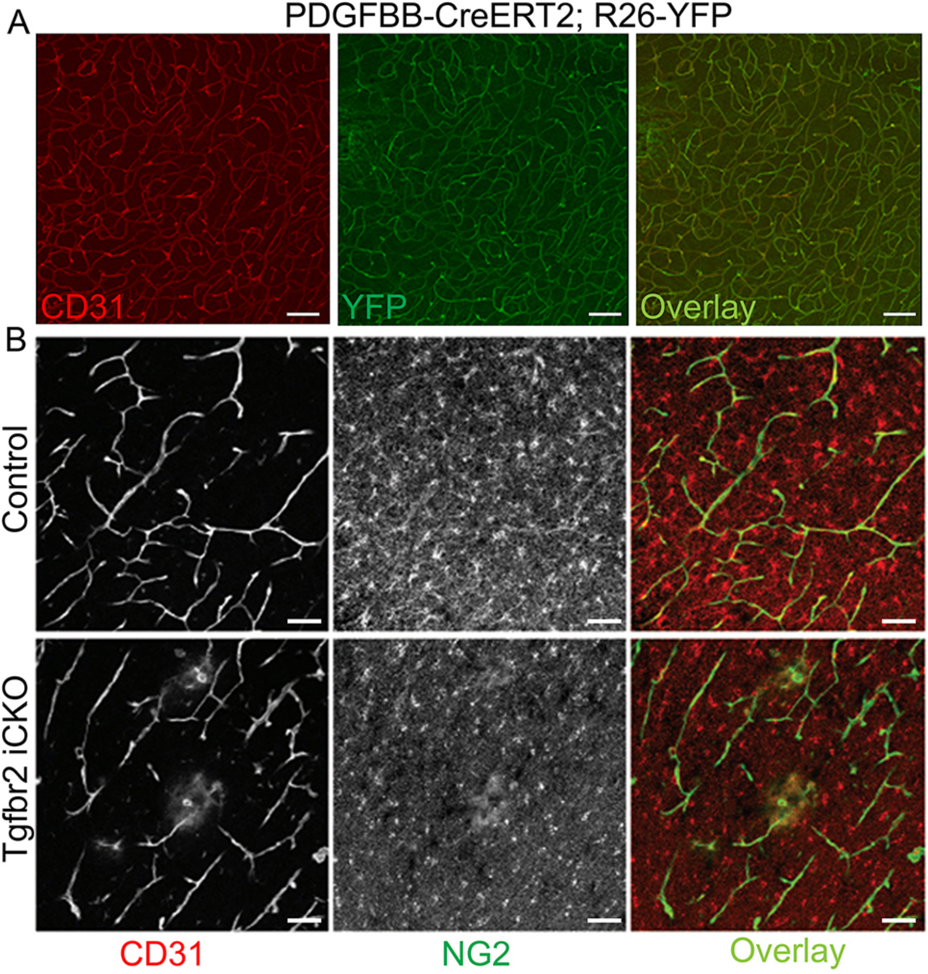
A
PDGFBB-CreERT2; R26-YFP
CD31
YFP
Overlay
B
Control
Tgfbr2 iCKO
CD31
NG2
Overlay

Tiwary et al., Supp Figure S8

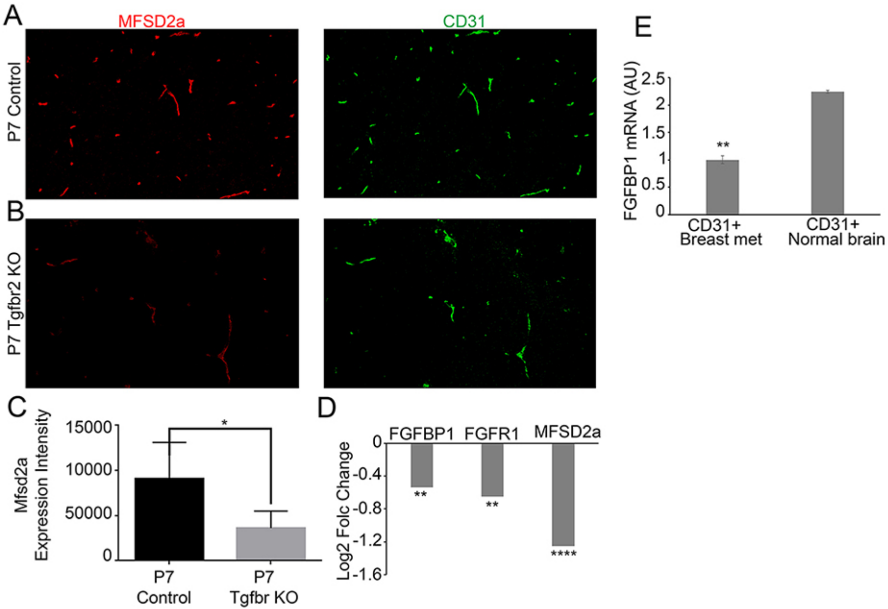

Supplement: Supplementary file 1 — Supplemental Figures and Legends [file 41598_2018_26636_MOESM1_ESM.pdf]
